# Mesoporous gold sponges: electric charge-assisted seed mediated synthesis and application as surface-enhanced Raman scattering substrates

**DOI:** 10.1038/srep16137

**Published:** 2015-11-05

**Authors:** Zao Yi, Jiangshan Luo, Xiulan Tan, Yong Yi, Weitang Yao, Xiaoli Kang, Xin Ye, Wenkun Zhu, Tao Duan, Yougen Yi, Yongjian Tang

**Affiliations:** 1 Joint Laboratory for Extreme Conditions Matter Properties, Southwest University of Science and Technology and Research Center of Laser Fusion, CAEP, Mianyang 621900, China; 2 Research Center of Laser Fusion, China Academy of Engineering Physics, Mianyang 621900, China; 3 College of Physics and Electronics, Central South University, Changsha 410083, China

## Abstract

Mesoporous gold sponges were prepared using 4-dimethylaminopyridine (DMAP)-stabilized Au seeds. This is a general process, which involves a simple template-free method, room temperature reduction of HAuCl_4_·4H_2_O with hydroxylamine. The formation process of mesoporous gold sponges could be accounted for the electrostatic interaction (the small Au nanoparticles (~3 nm) and the positively charged DMAP-stabilized Au seeds) and Ostwald ripening process. The mesoporous gold sponges had appeared to undergo electrostatic adsorption initially, sequentially linear aggregation, welding and Ostwald ripening, then, they randomly cross link into self-supporting, three-dimensional networks with time. The mesoporous gold sponges exhibit higher surface area than the literature. In addition, application of the spongelike networks as an active material for surface-enhanced Raman scattering has been investigated by employing 4-aminothiophenol (4-ATP) molecules as a probe.

In recent years, porous sponge-like noble (Au and Ag) metals with a high surface area, large pore volume, thermal conductivity and gas permeability, have received much attention for their applications in fuel cells, sensors, electrodes, catalysis and actuators^1^,^2^,^3^,^4^,^5^,^6^. To optimize and extend the applications of these noble metal nanoparticles, a number of synthetic methodologies have been developed. For example, dealloying process involves the selective dissolution of one or more components from a metallic solid solution such as Cu/Au and Ag/Au alloys^7^,^8^,^9^,^10^. On the other hand, hard inorganic templates (e.g., porous silica, anodic alumina membranes and aerogels)^11^,^12^,^13^ and soft organic templates (e.g., surfactants and polymers)^14^,^15^ have also been widely used in the fabrication of porous noble metals. However, these methods involve cumbersome procedures for the removal of the sacrificial metal or organic templates using concentrated acids or calcinations. Nevertheless, these template-based methods were routinely used to make porous noble metals with very low surface area (<2 m^2^/g)^16^. The porous sponge prepared by dextran templating and calcinations can be of arbitary macroscopic size and shape, but both the filaments and pores are in the micron size range, with the maxium BET surface area of about 0.4 m^2^/g^17^. Recently hydrothermal^18^,^19^, pH-controlled reduction by glucose^20^, evaporation-induced colloidal crystal growth^21^, electrochemical^22^ and microwave^23^ were also used to make porous noble metal nanostructures. In a different approach, fusion of preformed noble metal nanoparticles into network structures was also being explored recently^24^,^25^,^26^. And at the synthesis, electrostatic action process is extremely interesting to prepare three-dimensional (3D) porous noble metal nanostructures^27^,^28^.

Although a variety of mesoporous gold sponges are now available (All of these processes reported so far are either multistep, nonscalable processes and/or restricted to one or two metals or yield only a very low surface area.), yet it still remains as a challenge to fabricate porous gold sponges through a facile process under moderate conditions. Seed-mediated process is extremely interesting and attractive in the synthesis of different morphologies Au nanomaterials, such as nanorods^29^,^30^,^31^, spherical nanoparticles^32^, cubes^33^,^34^, bipyramids^35^ and branched particles^36^,^37^. However, the manufacture of mesoporous gold sponges with seed-mediated crystal growth has not been reported.

Herein, we demonstrated a facile seed-mediated procedure (using DMAP-stabilized Au seeds) to prepare 3D mesoporous gold sponges with high surface area by adding hydroxylamine at room temperature. The Au seeds are stabilized by DMAP and as a result are positively charged. The process involves a simple template-free method, room temperature reduction of HAuCl_4_·4H_2_O in water and is, therefore, scalable to any amount. To the best of our knowledge, these are the highest surface areas reported (45.53 m^2^/g) so far for any self-supported mesoporous gold sponge in one simple, template-free method. The test of surface-enhanced Raman scattering (SERS) from 4-aminothiophenol (4-ATP) molecules shows that the prepared mesoporous gold sponges are an active SERS substrate. These mesoporous gold sponges have large numbers of electromagnetic “hot-spots” because of the particle junctions, the high surface area and the sharp nanotips of the broken ligaments. Hence, seed-mediated preparation of SERS substrates described in this work has potential applications in chemical and biological analysis as well as medical detection.

## Results and Discussion

**Figure 1:**
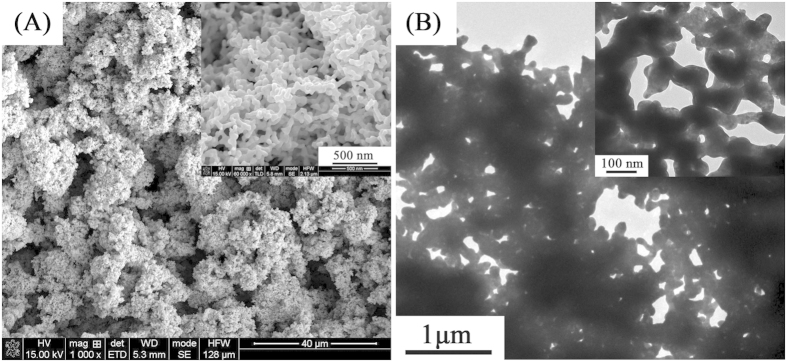
The morphology of mesoporous gold sponges: (**A**) SEM; (**B**) TEM. The inset in the SEM and TEM images represent the higher magnification.

**Figure 2:**
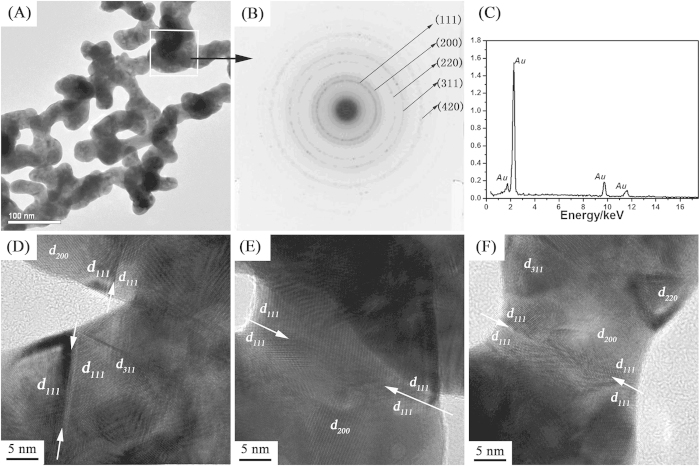
(**A**) TEM observation of mesoporous gold sponges; (**B**) SAED pattern of mesoporous gold sponges; (**C**) EDX of mesoporous gold sponges; (**D**–**F**) HRTEM images for mesoporous gold sponges. Lattice fringes are indicated with respective d-spacing symbols and some grain boundaries with arrow pairs.

The nanostructures of the fabricated mesoporous gold sponges through seed mediated method were examined by SEM and TEM images (Fig. 1). Figure 1A show typical SEM images of the gold sponge fabricated in this study at 1000 × and 60,000 × magnifications. The high magnification SEM image (inset of the Fig. 1A) reveals that the nanoparticles and chain-like fibers are interconnected, forming 3D networks. The diameter of the formed randomly sponge structures vary from 10 to 60 of nanometers (Supporting Information, Figure S1). A typical optical image of gold sponge in water is shown (Supporting Information, Figure S2), where we know the samples are the russet precipitate. The conversion yield of the as-synthesized gold sponge should be high (Here, in our experiment, the quality of gold sponge is 0.44 g by 1 g HAuCl_4_·4H_2_O, the conversion is about 93%), which is indicated by a colorless solution phase (i.e., no freestanding GNPs) after our seed mediated treatment. To further investigate the morphology of these mesoporous sponges, we show some TEM images in Fig. 1B. The TEM images further confirm these mesoporous sponges are netlike morphology. The image reveals the mesoporous sponges have a closely welded assembly with remarkable inter-particle contact regions. To further confirm the structure of the obtained products, typical SAED pattern of the products is shown (Fig. 2B). The SAED shows that the resultant sponges belong to the face-centered cubic crystal structure of metallic Au^38^. The calculated distances are 0.239, 0.208, 0.147 and 0.124 nm, which agrees well with the distances reported in literature for (111), (200), (220) and (311) planes of metallic Au, respectively (JCPDS File No. 04–0783 from ASTM). A typical XRD pattern is shown (Supporting Information, Figure S3), in which all the peaks could be indexed to the corresponding phase Au. Judging from the XRD patterns, the products have very high crystallinity. Energy dispersive X-ray spectroscopy (EDX) shows that the particles are composed of only Au metal as shown (Fig. 2C). The higher magnification TEM images (Fig. 2C,D) show well defined grain boundaries between the individual, crystalline NPs, oriented along random crystallographic axes. Lattice fringes are indicated with respective d-spacing symbols and some grain boundaries with arrow pairs. Here we find an interesting phenomenon that {111} planes appear at the both sides of grain boundaries. The reason is discussed in the growth mechanism part of the text. In this regard, the nanowires in the Au sponges as a basic skeletal support for the 3D network, while the junctions help to interconnect all the Au nanowires to form a complete and robust structure. It should be mentioned that the resultant sponge structures are stable under ambient condition for at least 3 months hitherto and almost kept unaltered even after vigorous sonication. Therefore, the present new approach promises a large scale production of Au sponge (refer to Fig. 1) via seed mediated treatment.

**Figure 3:**
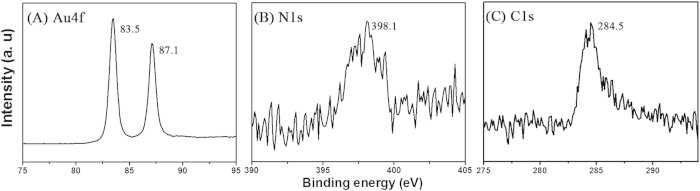
High resolution XPS spectra details collected from mesoporous gold sponges: (**A**) Au4f; (**B**) N1s; (**C**) C1s.

**Figure 4:**
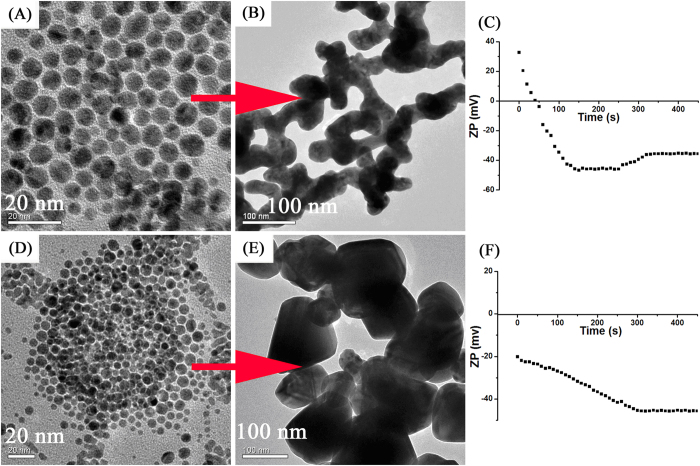
(**A**) TEM image of DMAP-stabilized Au seeds; (**B**) TEM image of the Au sponges prepared by DMAP-stabilized Au seeds growth solutions; (**C**) Zeta potential of Au sponges prepared in the DMAP-stabilized Au seeds growth solution with different reaction time; (**D**) TEM image of common Au seeds; (**E**) TEM image of the Au nanocrystals prepared by common Au seeds growth solutions; (**F**) Zeta potential of Au nanocrystals prepared in the common Au seeds growth solution with different reaction time.

XPS spectra can provide us with further information regarding the chemical state of the mesoporous gold sponges. Figure 3 presents the high-resolution XPS spectra displaying the Au 4f (Fig. 3A), N1s (Fig. 3B) and C1s (Fig. 3C). In agreement with expected results, the XPS survey scan has peaks indicating the presence of Au, C and N atoms from the mesoporous gold sponges (Supporting Information, Figure S4). Figure. 3A displays the XPS spectrum of the Au 4f region. The Au 4f7/2 and Au 4f5/2 peaks occur at a binding energy (BE) of 83.5 and 87.1 eV, respectively. According to previous literatures, while the Au 4f7/2 (84.0 eV) and Au 4f5/2 (87.7 eV) peaks could be assigned to metallic Au [19]. Additionally, here, it should be noted that the Au 4f peaks of mesoporous gold sponge are shifted by 0.5 eV toward lower BE. The previous report^39^ demonstrated that the analysis of BE shifts was useful to investigate the electrical interaction between Ag and Pd atoms. The lower shift of binding energy may be related to several factors. First, the surface of mesoporous gold sponge may be negatively charged, as shown the Fig. 4C. Second, the contact of mesoporous gold sponges and DMAP can induce the redistribution of the electron density. The main peaks of N1s at 398.1 eV and C1s at 284.5 eV can be assigned to surface-adsorbed DMAP^18^,^40^. On the basis of the above XPS analysis, it is clear that DMAP-stabilized Au seeds mediated process is an essential condition to activate surface reactivity of GNPs and thus to initiate 3D networking. Since it is still adsorbed on the Au sponges after particle aggregation, the DMAP also serves as a protecting reagent for the product.

As a further confirmation, we have also carried out normal reflux experiments under a uniform growth solution using the common Au seeds. Figure. 4 shows the TEM images of the nanoparticles prepared in the different Au seeds growth solutions: (A–B) DMAP-stabilized Au seeds and (D–F) common Au seeds. Zeta potential at DMAP-stabilized Au seeds growth solution (C) and common Au seeds growth solution (D). The solution is maintained at the same pH (~7). The Au seeds are stabilized by DMAP and as a result are positively charged, average z-potential of 32.7 mV (five measurements on three different transfer experiments, as show Fig. 4C at the reaction of beginning). The charge originates from the electron rearrangement across the pyridine aromatic ring to form an adsorbing charged species when exposed to water^38^. The change in zeta potential of Au sponges prepared in the DMAP-stabilized Au seeds growth solution with time (reaction time) is shown in Fig. 4(C). The zeta potential was 32.7 mV for the 0 s reaction sample which further decreased to −35.6 mV for the 450 s reaction sample. It can be observed that there was charge stabilization from 120 s to 250 s, with the charge stabilized about −45 mV. Also observably to observe was the increase in zeta potential after 250 s of interaction due to decreased stability of the system, which may be due to the GNPs randomly cross link into self-supporting, 3D networks. The mesoporous gold sponges begin appeared at 250 s. The stability of nanoparticles is determined by the surface charge density that the increase of surface charge density can decrease the tendency of aggregation and vice versa. Minor change in the measured zeta potential sometimes may indicate remarkable change in the surface charge density^41^. The UV-Vis spectra of the Au seeds solution were also recorded (Supporting Information S5). The absorbance of the common Au seeds solution is red-shifted about 13 nm compared with DMAP-stabilized Au seeds solution. The surface electric charge can contribute to this shift^42^. To explain the importance of the surface charge, common Au seeds have been used. The common Au seeds are negative charged, average z-potential of −20.1 mV (as show Fig. 4F). As shown in Fig. 4 (D–F), the nanoparticles are quasi-spherical when the reaction was performed under conditions similar to those used in Fig. 4(A,B) except that the seeds are common Au seeds. So, the seed mediated process of mesoporous gold sponge was strongly dependent on the electric charge of the Au seeds.

**Figure 5:**
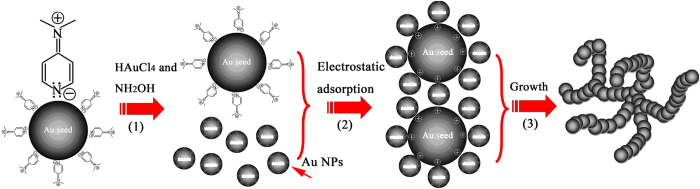
Schematic illustration of the seed mediated method to synthesize the mesoporous gold sponges.

The formation process of mesoporous gold sponge is shown in Fig. 5. This formation mechanism for mesoporous gold sponge is interesting, which takes advantage of electrostatic interaction and Ostwald ripening process. Starting from the reaction (Step 1), hydroxylamine promoted the rapid reduction of HAuCl_4_ and thus rapid formation of small Au particles (~3 nm) in the growth solution^43^. The small Au particles are negative charged, which results in the nanoparticles infiltrating and binding to the DMAP-stabilized Au seeds (Step 2), and then followed by 3D network (Step 3). Here, the DMAP-stabilized Au seeds have had the function of template. Because of different surface energy between the planes, the DMAP in solution served as capping agent and preferentially adsorbed the {111} planes of these Au seeds and decreased the surface energy of these planes^44^. As the DMAP molecule adsorbs on Au forming a protective shell of around 1 nm thickness^45^, thereby these small negative charged Au particles will bind to the {111} planes of DMAP-stabilized Au seeds through electrostatic adsorption, and the chain begin appearing. The mechanism can explain the phenomenon of the HRTEM image (Fig. 2B and Supporting Information, Figure S4). Some small seeds were dissolved to atoms and then the larger chains grew at the expense of the smaller ones through an Ostwald ripening process promoted by the continuous reflux because of the different surface energy between the large and small GNPs. The Au chains grow to NWs forming 3D mesoporous gold sponge architectures. So, the formation process of mesoporous gold sponge at different reaction time can be explained as a continuous process of nucleation - adsorption - growth - branching process.

**Figure 6:**
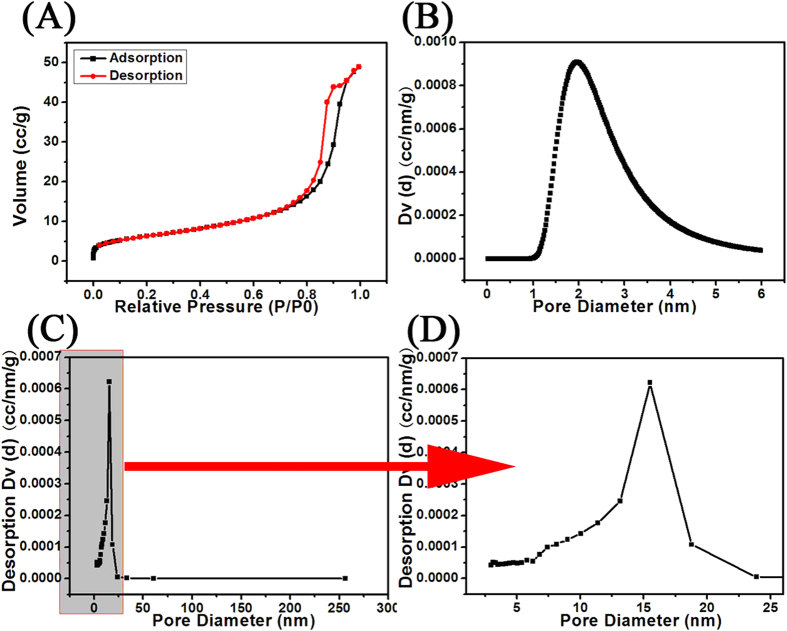
(**A**) Nitrogen adsorption/desorption isotherms (at 77 K) of the mesoporous gold sponges. (**B**) DA pore size distribution plot of the mesoporous gold sponges. (**C**,**D**) BJH pore size distribution plot of the mesoporous gold sponges.

On the basis of the above discussion, the mesoporous gold sponge constructed with 3D networks should have a large quantity of pores. To investigate the porous structure of the mesoporous gold sponge, a N_2_ adsorption/desorption isotherm was obtained. The nitrogen adsorption/desorption isotherm (at 77 K) of the mesoporous gold sponge is shown in Fig. 6 (A). The isotherm corresponding to the gold sponge is of type IV according to Brunauer-Deming-Deming- Teller (BDDT) classification with two capillary condensation steps, implying the bimodal pore-size distributions in the mesoporous and macroporous region. The hysteresis loop in lower relative pressure range (0.8 < P/P_0_ < 0.95) is related to finer intra-aggregated pore within the primary agglomerated particles, and that in higher relative pressure range (0.95 < P/P_0_ < 1) is associated with larger inter-aggregated pore between the secondary aggregated particles. The surface area of mesoporous gold sponge measured using the Brunauer-Emmett-Teller (BET) method shows 45.53 m^2^/g. Pore size distribution and pore characteristics were analyzed using Dubinin-Astakhov (DA) and Barrett-Joyner-Halenda (BJH) methods. Figure 6 (B) shows the DA plot of the mesoporous gold sponge. The equation fits the calculated data to experimental isotherm by varying the parameters in which the average adsorption energy is directly related to average pore diameter, and other parameter controls the width of the resulting pore size distribution. The pore radius obtained from the DA model in our studies comes out to be 2.3 nm. Pore size distribution plot of the mesoporous gold sponge by using the BJH model is shown in the Fig. 6(C,D). As can be seen the pore radius of the gold sponge spread in the range of 6–20 nm with the peak pore radius centered at 15.5 nm. The pore size obtained by the BJH model lies in the range for the pore size in mesoporous materials. The powders contain small mesopores (peak pore at 2.3 nm, as shown the Fig. 6 (B)) and larger mesopores with a peak pore diameter of ca. 15.5 nm (as shown the Fig. 6 (C,D)). The smaller mesopores reflects porosity within the primary agglomerated particles, while larger mesopores can be related to the pores formed between aggregated Au nanowires. The lack of plateau on the adsorption isotherm at the relative pressures approaching unity (which resembles type II isotherm curve) indicates the presence of macropores among aggregated Au nanowires, which is manifested by tailing of the pore-size distribution curve in direction of large pore sizes. This macroporous structure can be directly observed on the SEM images of mesoporous gold sponge shown in Fig. 1, which cannot be accessed by N_2_ adsorption-desorption analysis. In Table 1 several approaches for the preparation of mesoporous Gold sponges and their resulting surface areas are summarized. To the best of our knowledge, these are the highest surface areas reported so far for any self-supported mesoporous gold sponge in one simple, template-free method in our experiment. Since the mesoporous gold sponges were formed through fusion of metal nanoparticles/clusters (of size 3 nm Au seeds) emerged during the nucleation, they were expected to have surface roughness at the nanoscale which would contribute to their high BET surface area.

**Figure 7:**
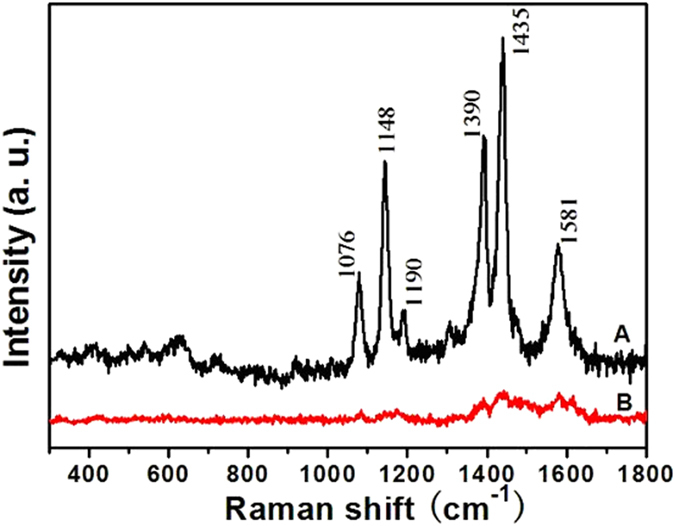
Raman spectra of 4-ATP (1 × 10^−6^ mol/L) on different substrates: (**A**) mesoporous gold sponges; (**B**) quasi-spherical gold nanoparticle.

Promoted by the superior sensitivity and molecular specificity, surface enhanced Raman scattering has been a powerful tool to label the bond vibrations of molecules. Chemical effect (CE) and electromagnetic (EM) effect were generally accepted as two major mechanisms responsible for the SERS activity with Au as substrate. An accepted reasonable explanation is that electromagnetic fields can be localized in the interparticle regions of investigated clusters, resulting in prominently enhanced absorption and other optical properties. As found, close-packed Au/Ag bimetallic hollow nanospheres films can be used as an effective active substrate for SERS application with large electromagnetic field enhanced factors. The effect was believed to originate from SERS-active sites at the junction positions among sharp edges, nanoscale roughness and hollow nanostructured aggregate of the Au/Ag bimetallic hollow nanospheres^46^. For our mesoporous gold sponge, there exist a large number of junction regions and hollow nanostructured in the mesoporous gold sponge. Therefore, according to previous theoretical and experimental investigations^46^,^47^, the mesoporous gold sponge can be expected to exhibit intense local electromagnetic (EM) field enhancement behavior and can serve as a SERS material for molecular sensing. Here, to evaluate the SERS enhancement ability of mesoporous gold sponge prepared by the present method, we compare a SERS spectrum of 4-ATP on mesoporous gold sponge with that on spherical gold nanoparticle. For SERS spectra measurements, a 10 μL droplet of 4-ATP ethanol solution (1.0 × 10^−6^ mol/L) was dropped on the samples using an accurate pipette and then dried in air at ambient temperature to obtain a uniform molecule deposition over an area of about 10 mm^2^. Figure 7 (A,B) compares the SERS spectrum of 4-ATP on the mesoporous gold sponge and quasi-spherical gold nanoparticle, respectively. Two sets of bands were observed on the SERS spectra of 4-ATP on the mesoporous gold sponge; one set is located at 1076 and 1190 cm^−1^, which are assigned to the a_1_ vibration modes, and the other set is located at 1148, 1390, 1435, and 1581 cm^−1^, which are assigned to the b_2_ vibration modes^48^. To determine the enhancement effect (EF) of 4-ATP on the nanoparticles quantitatively, the EF values of 4-ATP in the nanoparticles are calculated with the following expression (the detailed calculation could be seen in the Supporting Information):


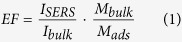


Then the EF for the mesoporous gold sponge and quasi-spherical gold nanoparticles were roughly estimated by comparing the peak intensity at 1076 cm^−1^ to 6.84 × 10^4^ and 3.36 × 10^3^, respectively. The SERS signal of 4-ATP on the mesoporous gold sponge is about 20 times stronger than that on the quasi-spherical gold nanoparticles. We believe the SERS enhancement is mainly due to the local electromagnetic field enhancement. In our case, the junction in the branched chains may serve as the hot sites. In the mesoporous gold sponge, the ligaments are welded by many GNPs, resulting in an abundance of grain boundaries, which provides a larger number of high activity sites for SERS sensors. The as-prepared mesoporous gold sponge, unlike quasi-spherical nanoparticle SERS substrates that rely on interparticle fusion, have a great number of particle junctions, which can act as “hot sites” for surface plasma and cause “rough surface” EM enhancement^49^. The plasmon coupling of mesoporous gold sponge at these hot spots will produce a very intense local EM field and consequently strong SERS signals. On the other hand, the effect of nanopores on the EM enhancement should be considered. These nanopores can result in stronger EM coupling between the vertically standing spongelike networks films that are parallel to the incident lasers, which can generate plenty of “hotspots” by the local optical coupling with suitable gap width (Fig. 1). Moreover, the sharp nanotips of the broken ligaments (Fig. 1A) can also provide strongly localized EM fields for ultrahigh SERS enhancements^49^ because of the additional lightning rod effect^50^.

## Conclusions

In this paper, we presented a seed mediated method to fabricate mesoporous gold sponges with a highly surface area (45.53 m^2^/g). The formation process of mesoporous gold sponges could be accounted for by the electrostatic interaction (the small GNPs (~3 nm) and the positively charged DMAP-stabilized Au seeds) and Ostwald ripening process. Mesoporous gold sponges were shown as good substrates for SERS activity. The junction regions, the hollow nanostructured and the sharp nanotips of the broken ligaments in the mesoporous gold sponge were act as electromagnetic “hot-spots”. This promising method is expected to be suitable to other 3D porous networks nanomaterials of noble metals.

## Materials and Methods

### Chemicals

All of the chemicals were used as received without further purification: HAuCl_4_·3H_2_O, NaBH_4_, TOAB, DMAP, Na_3_Cit and NH_2_OH·HCl from Sigma-Aldrich. Water was distilled using a water purification system (Barnstead D12651).

### Preparation of DMAP-stabilized Au seeds

DMAP-stabilized Au seeds were synthesized using the phase transfer procedure reported by Gittins^51^. Briefly, 30 mL (30 mM) aqueous solution of HAuCl_4_·4H_2_O was added to 80 mL (25 mM) of TOAB solution in toluene. Then, 25 mL of 0.4 M aqueous NaBH_4_ solution was added to the mixture under stirring to cause a reduction of HAuCl_4_·3H_2_O. After 30 min, the two phases were separated and the toluene phase was subsequently washed with 0.1M H_2_SO_4_, 0.1M NaOH, and H_2_O (three times), and then dried over anhydrous Na_2_SO_4_. To prepare positive-charged Au nanoparticles (GNPs), an aqueous 0.1M DMAP solution (5 mL) was added to the as-made GNPs of the same volume (5 mL). Then TOAB, which stabilized the surfaces of GNPs in toluene, spontaneously substituted for DMAP and DMAP-terminated GNPs moved to water phase (20 mL). In order to improve the substitution to DMAP for TOAB, the mixture solutions were gently agitated for several times.

### Preparation of common Au seeds

Common Au seeds were synthesized using the procedure reported by Jana *et al.*^52^ 25 mL HAuCl_4_ aqueous solution was taken in a screw-capped glass bottle and Na_3_Cit was added in it. Then, 0.6 mL of aqueous NaBH_4_ (0.1 mM) solution was added under constant stirring giving rise to a ruby red color to the solution with a final [HAuCl_4_] = [Na_3_Cit] = 0.5 mM, which acts as a seed solution.

### Seed mediated growth

Typically, 2 ml DMAP-stabilized Au seeds solution (or Common Au seeds solution) was added to 50 mL HAuCl_4_ aqueous solution (20 mM) and mixed. Then 50 mL NH_2_OH·HCl aqueous solution (40 mM) was added to the mixed solution quickly. The stirring was continued for about 10 min until the entire solution became colorless. The russet precipitates were washed with distilled water and dried at room temperature.

In a similar method, Ag nanosponges were prepared by adding 2 ml DMAP-stabilized Au seeds solution into 50 mL AgNO_3_ aqueous solution (20 mM). Then 50 mL NH_2_OH·HCl aqueous solution (40 mM) was added to the mixed solution quickly. The typical SEM image and EDX of Ag sponge are shown the Supporting Information (Figure S6).

### Characterization

A JEM-2010 transmission electron microscope (TEM) was used to observe the nanoparticles. The Scanning electron microscopy (SEM) images were recorded by using a Leica Cambridge S440 field emission scanning electron microscope with an accelerating voltage of 5.0 kV. Energy dispersive X-ray spectra (EDX) were recorded on an Oxford INCA energy spectroscope. X-ray photoelectron spectroscopy (XPS) measurements were carried out on an ESCALAB-MKII spectrometer (VG Co., UK) with Al Kα X-ray radiation as the X-ray source for excitation. Elemental analysis was performed on Flash EA 1112. X-ray diffraction patterns (XRD) were recorded on an X’Pert PRO X-ray diffractometer using Cu Kα (40 kV, 40 mA) radiation. XRD patterns have been measured in reflection mode. All UV-Vis-NIR spectra were recorded within a quartz cell of 1 cm optical length on a Perkin-Elmer Lambda 12 spectrophotometer. The Brunauer-Emmett-Teller (BET) specific surface area (SBET) of the powders (1 g) was analyzed by nitrogen adsorption in a Micromeritics ASAP 2020 nitrogen adsorption apparatus (USA). All the as-prepared samples were degassed at 100 °C prior to nitrogen adsorption measurements. The zeta potential of the synthesized nanoparticles was measured on a Malvern ZetasizerNano-ZS90 (Malvern Instruments Ltd., UK) at 25.0 ± 0.1 °C. Raman spectra were obtained with a Renishaw 2000 model confocal microscopy Raman spectrometer with a CCD detector and a holographic notch filter (Renishaw Ltd, Gloucestershire, U.K.). The microscope attachment was based on a Leica DMLM system, and an objective was used to focus the laser beam onto a spot with approximately 1 μm in diameter. Radiation of 514.5 nm from an air cooled argon ion laser (Spectra-Physics Model 163-C4260) was used for excitation. All of the spectra reported were the results of a single accumulation of 20 s.

## Additional Information

**How to cite this article**: Yi, Z. *et al.* Mesoporous gold sponges: electric charge-assisted seed mediated synthesis and application as surface-enhanced Raman scattering substrates. *Sci. Rep.* **5**, 16137; doi: 10.1038/srep16137 (2015).

## Supplementary Material

Supplementary Information

## Figures and Tables

**Table 1:** BET Surface Area Values of Mesoporous Gold Sponges Prepared by Several Techniques.

Method	Surface area [m^2^/g]	Ref.
Dealloying of complex white gold alloy	14.2 m^2^/g	3
Combustion synthesis with metal bistetrazolamine complexes	10.9 m^2^/g	5
Electrochemical dealloying and thermally annealed approaches	3.01–7.83 m^2^/g	6
Electrochemical dealloying of Ag_75_Au_25_	10–15 m^2^/g	9
Template-dealloying approach	23.6 m^2^/g	13
Template-free assembly of glucose stabilized NPs	11.9 m^2^/g	20
Sol-Gel Approaches	41 m^2^/g	26
