# Supplementary material for: Mesoporous gold sponges: electric charge-assisted seed mediated synthesis and application as surface-enhanced Raman scattering substrates
Source: Sci Rep. 2015 Nov 5;5:16137. doi: 10.1038/srep16137 (PMC4633612; doi:10.1038/srep16137)
Supplement: Supplementary Information [file srep16137-s1.doc]

**Electronic supplementary information**

**Mesoporous gold sponges: electric charge-assisted seed mediated synthesis and application as surface-enhanced Raman scattering substrates**

**Zao Yi ^1,2^, Jiangshan Luo ^2^, Xiulan Tan ^2^, Yong Yi^1^**[[1]](#footnote-2)***, Weitang Yao^1^, Xiaoli Kang^2^, Xin Ye^2^, Wenkun Zhu^1^, Tao Duan^1*^, Yougen Yi^3*^, Yongjian Tang^1,2^**

^1^Joint Laboratory for Extreme Conditions Matter Properties, Southwest University of Science and Technology and Research Center of Laser Fusion, CAEP, Mianyang 621900, China

^2^Research Center of Laser Fusion, China Academy of Engineering Physics, Mianyang 621900, China

^3^College of Physics and Electronics, Central South University, Changsha 410083, China


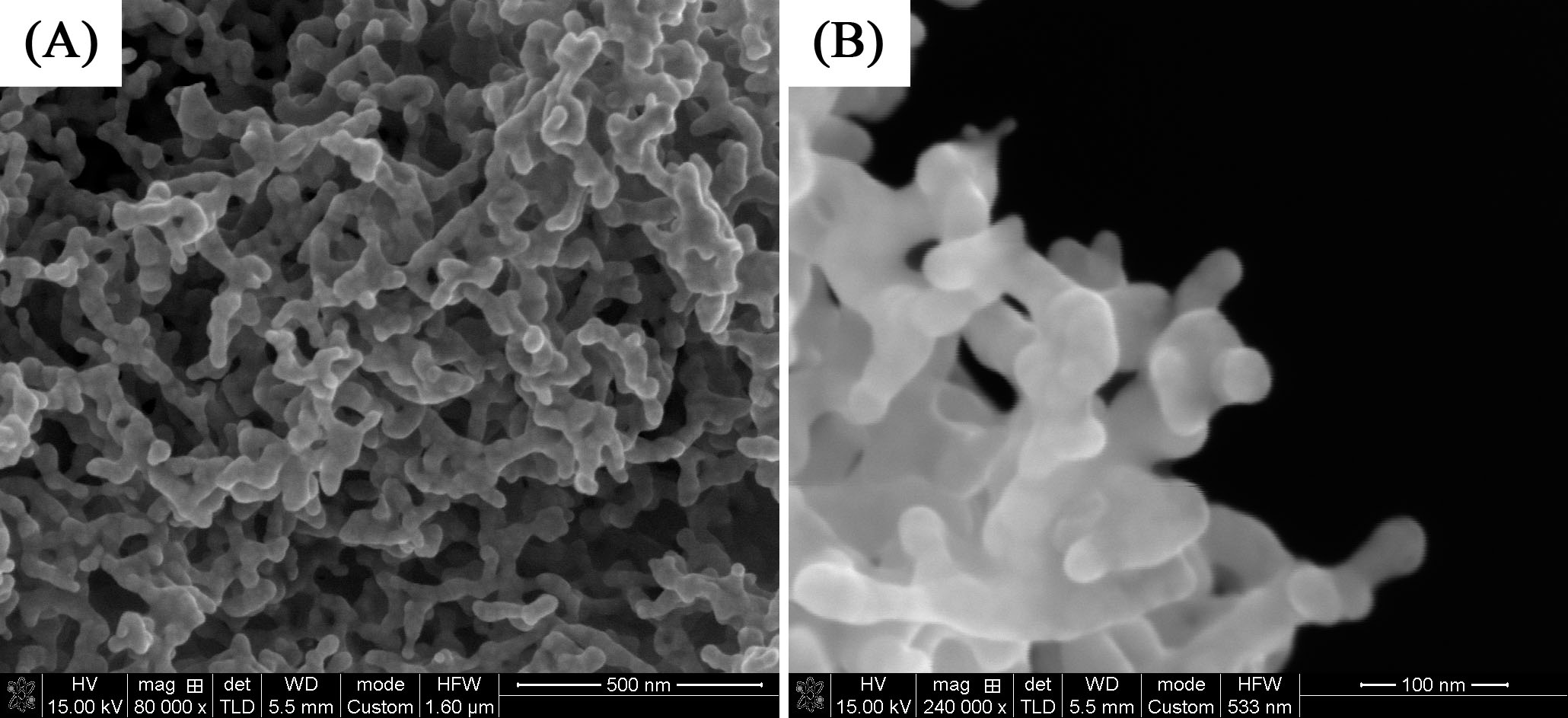


Figure S1: FESEM images showing mesoporous gold sponges with highly interconnected ligaments of size 10-60 nm.


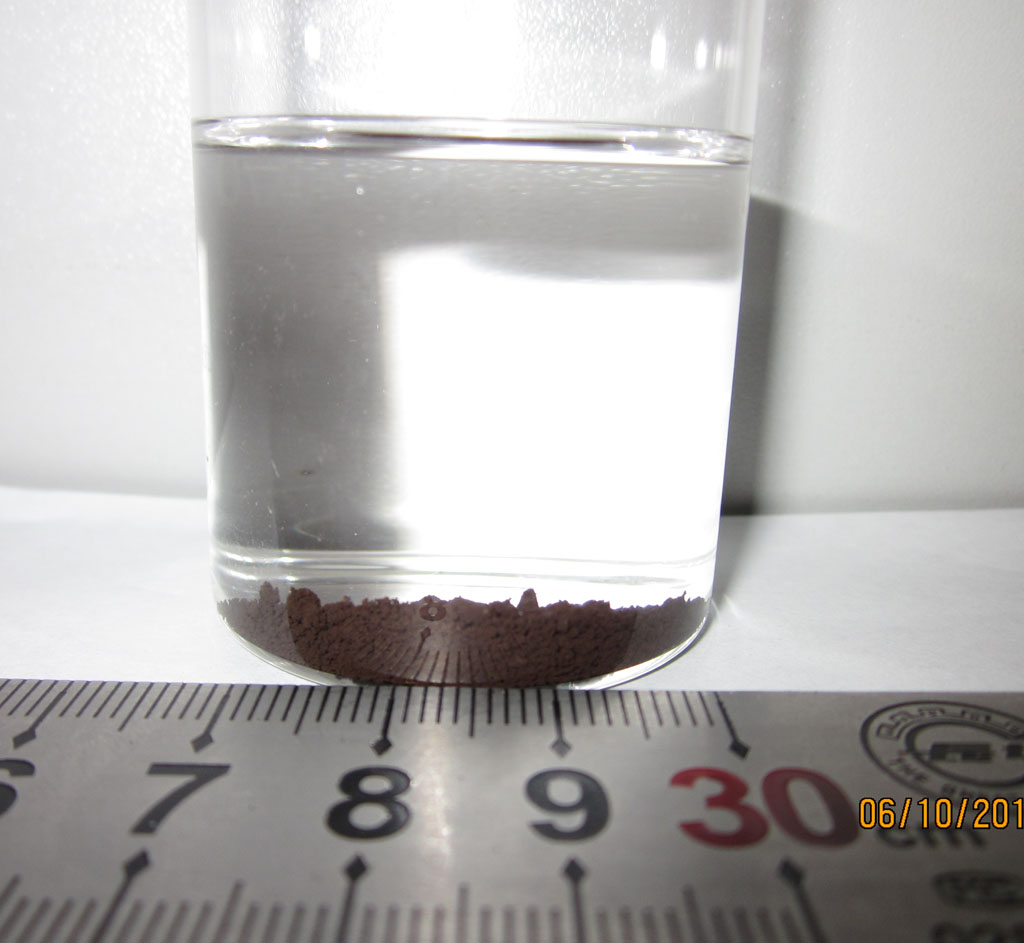


Figure S2: Optical image of the mesoporous gold sponges in water.


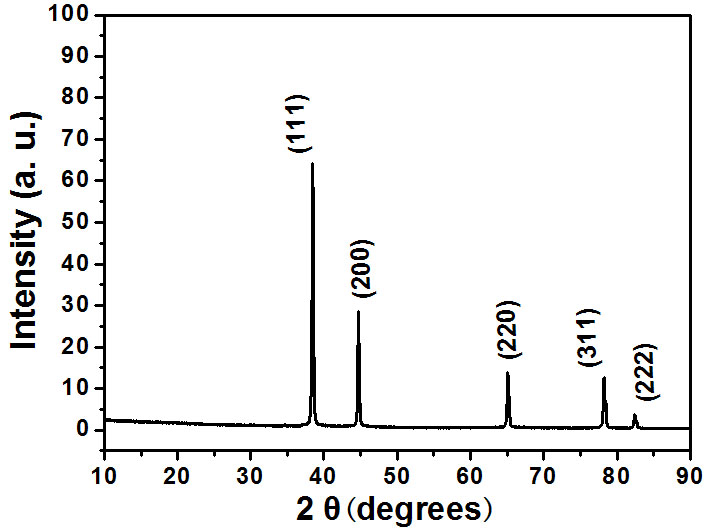


Figure S3: XRD of the mesoporous gold sponges


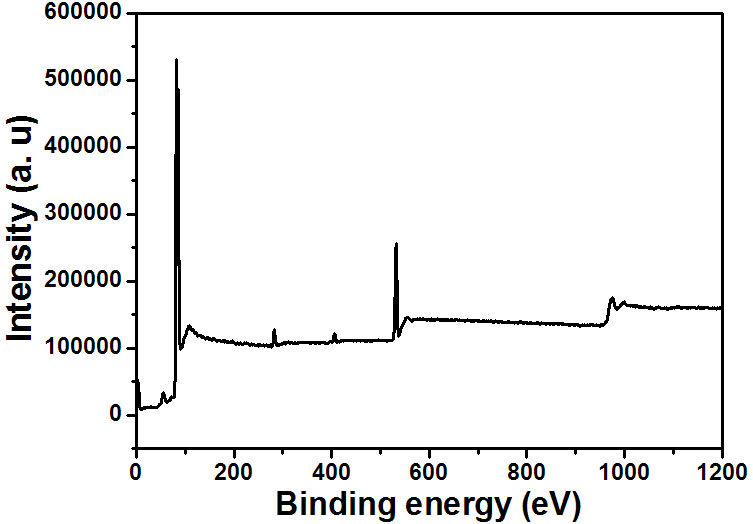


Figure S4: XPS survey spectra of the mesoporous gold sponges.


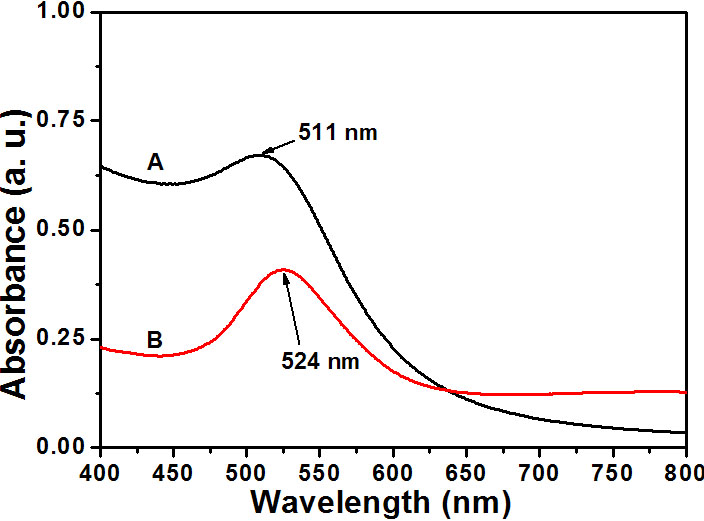


Figure S5: UV-Vis absorption spectra of the seeds solution: (A) DMAP-stabilized Au seeds; (B) commonly Au seeds.


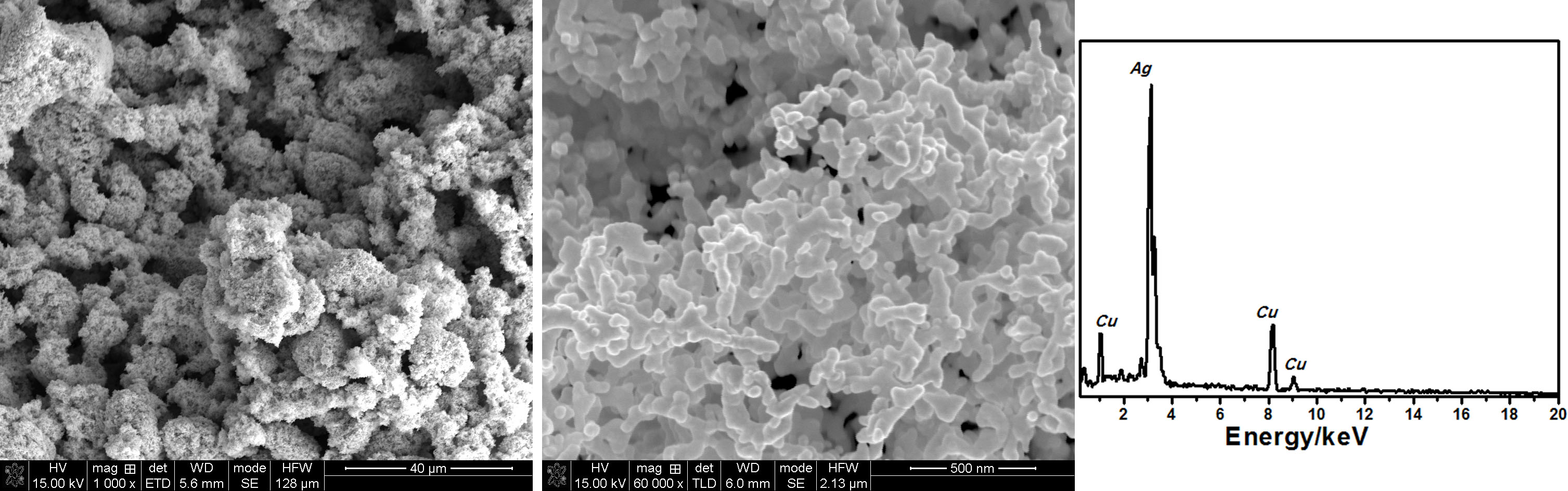


Figure S6: The morphology (SEM) and EDX of mesoporous Ag sponges.

**The calculation of EF is followed:**

To determine the enhancement effect of 4-ATP on the nanoparticles quantitatively, the enhancement factor (EF) values of 4-ATP in the nanoparticles is calculated the following expression:

$EF=\frac{I_{SERS}}{I_{bulk}}\bullet\frac{M_{bulk}}{M_{ads}}$ (1)

Where *I*_SERS_ stands for the intensity of a vibrational mode in the SERS spectrum of 4-ATP and *I*_bulk_ for that of solid sample. *M*_ads_ and *M*_bulk_ are the number of 4-ATP molecules adsorbed on the SERS substrate and bulk molecules illuminated by the laser light to obtain the corresponding SERS and ordinary Raman spectra, respectively. *M*_ads_ can be obtained according to the method proposed by Guo et al. ^1^, which is

$M_{ads}=N_{d}A_{laser}A_{N}/\sigma$ (2)

Where *N*_d_ is the number density of the nanoparticles, *A*_laser_ is the area of the focal spot of laser, *A*_N_ is the nanoparticles footprint area, and σ is the surface area occupied by an adsorbed 4-ATP molecule. *N*_d_ and *A*_N_ can be obtained from the SEM images in Fig. 1, and *A*_laser_ can be obtained from the diameter of the laser spot (~1 μm). According to the report by Kim ^2^, each 4-ATP molecule occupies ~0.20 nm^2^, indicating that σ can be adopted as ~0.20 nm^2^/ molecule. Then the total number of surface adsorbed molecules (*M*_ads_) within the illuminated laser spot can be obtained at 4.4 × 10^5^. *M*_bulk_ is the molecule number of the solid 4-ATP in the laser illumination volume. According to the report by Hu ^3^, *M*_bulk_ was calculated to be about 8.9 × 10^9^ within the illuminated laser light. Then, the EF for the mesoporous gold sponge and quasi-spherical gold nanoparticles were roughly estimated by comparing the peak intensity at 1076 cm^-1^ to 6.84 ×10^4^ and 3.36×10^3^, respectively.

**Reference**

1 Guo, S. J., Dong, S. J. & Wang, E. K. Rectangular silver nanorods: Controlled preparation, liquid− liquid interface assembly, and application in surface-enhanced Raman scattering. Crystal Growth & Design, 9, 372-377 (2009).

2 Kim, K. & Yoon, J. K. Raman scattering of 4-aminobenzenethiol sandwiched between Ag/Au nanoparticle and macroscopically smooth Au substrate. J. Phys. Chem. B 109 (44), 20731-20736 (2005).

3 Hu, X. G., Wang, T., Wang, L. & Dong, S. J. Surface-Enhanced Raman Scattering of 4-Aminothiophenol Self-Assembled Monolayers in Sandwich Structure with Nanoparticle Shape Dependence:  Off-Surface Plasmon Resonance Condition. J. Phys. Chem. C 111, 6962-6969 (2007).

1. ^^ Correspondence should be addressed to Yong Yi, Tao Duan and Yougen Yi

   Tel: 86-0816-2480830; Fax: 86-0816-2480830

   E-mail address: [myyz1984@csu.edu.cn](mailto:myyz1984@csu.edu.cn); Twcsu2013@csu.edu.cn; Yougenyi@ csu.edu.cn [↑](#footnote-ref-2)
